# Different Effects of Insulin-Like Growth Factor-1 and Insulin-Like Growth Factor-2 on Myogenic Differentiation of Human Mesenchymal Stem Cells

**DOI:** 10.1155/2017/8286248

**Published:** 2017-12-14

**Authors:** Doaa Aboalola, Victor K. M. Han

**Affiliations:** ^1^Department of Anatomy and Cell Biology, Schulich School of Medicine and Dentistry, University of Western Ontario, London, ON, Canada; ^2^Children's Health Research Institute, University of Western Ontario, London, ON, Canada; ^3^Lawson Health Research Institute, University of Western Ontario, London, ON, Canada; ^4^King Abdullah International Medical Research Center, National Guard Health Affairs, Jeddah, Saudi Arabia; ^5^Department of Paediatrics, Schulich School of Medicine and Dentistry, University of Western Ontario, London, ON, Canada

## Abstract

Insulin-like growth factors (IGFs) are critical components of the stem cell niche, as they regulate proliferation and differentiation of stem cells into different lineages, including skeletal muscle. We have previously reported that insulin-like growth factor binding protein-6 (IGFBP-6), which has high affinity for IGF-2, alters the differentiation process of placental mesenchymal stem cells (PMSCs) into skeletal muscle. In this study, we determined the roles of IGF-1 and IGF-2 and their interactions with IGFBP-6. We showed that IGF-1 increased IGFBP-6 levels within 24 hours but decreased after 3 days, while IGF-2 maintained higher levels of IGFBP-6 throughout myogenesis. IGF-1 increased IGFBP-6 in the early phase as a requirement for muscle commitment. In contrast, IGF-2 enhanced muscle differentiation as shown by the expression of muscle differentiation markers MyoD, MyoG, and MHC. IGF-1 and IGF-2 had different effects on muscle differentiation with IGF-1 promoting early commitment to muscle and IGF-2 promoting complete muscle differentiation. We also showed that PMSCs acquired increasing capacity to synthesize IGF-2 during muscle differentiation, and the capacity increased as the differentiation progressed suggesting an autocrine and/or paracrine effect. Additionally, we demonstrated that IGFBP-6 could enhance the muscle differentiation process in the absence of IGF-2.

## 1. Introduction

Insulin-like growth factor (IGF) system regulates cell growth, differentiation, migration, and cell survival through activation of several receptor-dependent signal transduction pathways [1]. The IGF family consists of two IGF ligands (IGF-1 and IGF-2), three cell surface receptors (IGF-1 and IGF-2 receptors and the insulin receptor), and six IGF binding proteins (IGFBPs) [1]. IGF-1 and IGF-2 are circulating peptides, which bind IGF-1R, a ligand-activated receptor tyrosine kinase, for their biological actions [1, 2]. IGFBPs act as carriers for IGFs in the circulation [3], protecting them from degradation [2, 4] and regulating the biological actions of IGFs by delivering them to specific tissues. The IGF family plays an important role in fetal and placental development by stimulating proliferation, differentiation, and survival of various types of placental cells [5]. IGFs are vital in cell growth, development, and cell-fate changes through several mitogen activation cascades [6–9]. The IGF family is also important in muscle development as IGFs maintain muscle cell viability, promote hypertrophy, and stimulate differentiation in cultured myoblasts [9]. The importance of IGFs and the IGF-1R in skeletal muscle development is demonstrated in the loss-of-function animal models. IGF-1R knockout mice die soon after birth as functional respiratory muscle is deficient and pups are unable to breathe [10, 11]. Furthermore, IGF-1 and IGF-2 are expressed by skeletal muscle cells during muscle repair in response to muscle injury and exercise [12, 13]. IGFs are the only factors known to promote both muscle cell proliferation and differentiation [13]. In response to muscle injury, adult skeletal muscle regenerates by expressing myogenic regulatory factors (MRFs) [13]. During myogenesis, committed muscle cells differentiate into the muscle lineage by expressing muscle commitment markers, Pax3 and Pax7, which in turn upregulate MyoD and myogenin [14]. After commitment, myoblasts fuse together and form multinucleated fibers that express myosin heavy chain (MHC) [14].

After muscle injury, IGF-1 enhances regeneration while inhibiting IGF-1 activity with neutralizing antibodies, reducing the number of regenerating myofibers *in vivo* [15]. IGF-2, which upregulates its own gene expression during myogenesis in a positive feedback loop [12], is expressed abundantly in the developing skeletal muscle and is the major growth factor for muscle growth, differentiation, and regeneration [12, 13]. When IGF-2 is inhibited, myogenesis does not occur [16]. In fact, IGF-2 is required to allow the continued recruitment of MyoD-associated proteins at the myogenin promoter [17]. Moreover, in cultured myoblasts, IGFs stimulate terminal differentiation through an autocrine pathway that is dependent on IGF-2 secretion [18].

IGFBPs also play a role in fetal and placental development as they are expressed during different stages of development in a time-specific and cell-specific manner. Although loss-of-function studies targeting single IGFBP genes have not yielded significant phenotypic changes in the fetus or placenta, it is thought that there are subtle changes in development which are possibly due to biologic compensation by other binding proteins or redundancy. IGFBP-6, a member of the IGF binding protein family, is expressed abundantly in developing muscle cells and is required for myogenesis [13]. It is a unique peptide among the IGFBPs due to its higher binding affinity to IGF-2 versus IGF-1 (~70- to 100-fold) [19–22]. The most commonly reported function of IGFBP-6 is the modulation of IGF-2 activity. IGFBP-6 binds IGF-2 in the circulation and prevents it from binding to the cell surface receptors and modulates IGF-2 bioavailability *in vitro* and *in vivo* [23, 24]. IGFBP-6 can also bind and localize IGF-2 at the cell surface, enhancing IGF-2 actions by delivering IGF-2 to the IGF-1R [24]. However, the mechanisms controlling the multiple actions of IGFBP-6 remain unclear. Previous studies in our lab have shown that both extracellular and intracellular IGFBP-6 are required for myogenesis. In this study, we determined the different biologic roles of IGF-1 and IGF-2 and whether IGFBP-6's impact on PMSC differentiation into skeletal muscle is dependent or independent of IGFs (particularly IGF-2).

## 2. Materials and Methods

### 2.1. Isolation of PMSCs

PMSC isolation and experiments were conducted in accordance with the approval from the Health Sciences Research Ethics Board of Western University. Informed consent was obtained from healthy women undergoing therapeutic termination of pregnancy, and the PMSCs used in this study were isolated from 15 weeks preterm placental tissues. After surgery, chorionic villi were dissected, washed, minced with surgical scissors and forceps, and then subjected to enzymatic digestion with collagenase IV (369 IU/mg), hyaluronidase (999 IU/mg) (Sigma-Aldrich, Oakville, ON), and DNase I (2000 IU/mg) (Hoffmann-La Roche, Mississauga, ON) for 10 minutes at room temperature, followed by 0.05% trypsin (Gibco/Invitrogen, Mississauga, ON) for 5 minutes at room temperature. The sample was then washed for 10 minutes with 10% FBS in DMEM/F12 medium, and the resulting single cell suspension was separated by density centrifugation over a Percoll (Sigma-Aldrich, Oakville, ON) discontinuous gradient using a modified protocol by Worton et al. [25, 26]. PMSCs isolated were validated for the expression of stem cell markers (CD73, CD105, and CD-117/c-Kit) and differentiation ability [26].

### 2.2. Muscle Differentiation and IGFs Treatment

Cells were plated in muscle growth media (fetal bovine serum 0.05 mL/mL, fetuin 50 *μ*g/mL, epidermal growth factor 10 ng/mL, basic fibroblast growth factor 1 ng/mL, insulin 10 *μ*g/mL, and dexamethasone 0.4 *μ*g/mL) for 48 hours before changing to the skeletal muscle differentiation media, which is a proprietary serum-free medium containing 10 *μ*g/mL insulin (PromoCell, Heidelberg, Germany) [26], with or without 100 ng/mL of either IGF-1 or IGF-2 (Cell Signaling Technology, Danvers, MA). IGFs were added every 3 days at the time of media change. Cells were grown in six-well plates in a standard tissue culture incubator at 37°C in 5% CO_2_.

### 2.3. Downregulation of IGF-2 Expression by siRNA

To silence the endogenous IGF-2 expression, siRNA against IGF-2 (Santa Cruz Biotechnology, Dallas, TX) was used. 8 *μ*L of Lipofectamine (Invitrogen, Mississauga, ON) with 8 *μ*L of either scrambled or IGF-2 siRNA was added to 100 *μ*L of DMEM/F12 media (Invitrogen, Mississauga, ON) for 40 minutes at room temperature. siRNA was added to the 70% confluent cells and incubated for 5 hours at 37°C. Muscle growth media (1.5 mL) was added for 48 hours, then changed to muscle differentiation media (2 mL) (PromoCell, Heidelberg, Germany).

### 2.4. IGFBP-6

Recombinant human IGFBP-6 (ProSpec, East Brunswick, NJ) was added to the media (375 ng/mL) every 3 days at the time of media change. The IGFBP-6 concentration was determined by a dose-response curve using PMSCs in muscle differentiation media [26].

### 2.5. Immunoblotting

Following PMSC culture, cell lysates containing 20 *μ*g of protein were added to 6x SDS gel loading buffer (1% *β*-mercaptoethanol, 1% SDS, 30% glycerol, 0.0012% bromophenol blue, Tris-HCl 0.28 M, pH 6.8). Samples were boiled for 5 minutes at 95°C, then placed on ice for 3 minutes, and centrifuged at 3000 rpm for 20 seconds before loading. Samples were resolved by molecular weight using 10% SDS polyacrylamide gels transferred onto polyvinylidene fluoride (PVDF) membranes (Bio-Rad, Hercules, CA) using a Trans-Blot Turbo (Bio-Rad, Hercules, CA) with an optimized protocol depending on protein size. Membranes were blocked with 5% nonfat dry milk, gently shaking for 1 hour at room temperature in Tris-HCl buffered saline pH 8.0 with 0.1% Tween-20 (TBS-T). Blots were then washed with TBS-T (3 times for 10 minutes) followed by incubation at 4°C overnight with specific primary antibodies in 5% BSA or 5% nonfat dry milk in TBS-T following the manufacturer's protocol. Then, membranes were washed and incubated for 1 hour at room temperature with the corresponding secondary HRP-conjugated antibody. Resolved protein bands were detected using chemiluminescence, and images were taken using the VersaDoc Imager (Bio-Rad, Hercules, CA) [26].

### 2.6. Immunocytochemistry

PMSCs were grown on glass coverslips, stained with primary antibodies (1 : 100), and incubated at 4°C overnight. Primary antibodies were washed using 0.1% Tween-20 in PBS (3 times for 5 minutes); cells were then incubated in the dark with the corresponding secondary Alexa Flour conjugated antibody (1 : 200). The secondary antibody was washed using 0.1% Tween-20 in PBS (3 times for 5 minutes), and the nuclear stain was added for 7 minutes and then rinsed. The cover slips were mounted and images were taken using a confocal microscope (Zeiss, Germany) [26].

### 2.7. Antibodies

To detect stem cell-associated potency markers, antibodies for OCT4 (N-19: sc-8628) (Santa Cruz Biotechnology, Dallas, TX), SOX2 (2683-1) (Epitomics, Burlington, ON), and Nanog (3369-1) (Epitomics, Burlington, ON, Canada) were used. To detect markers of muscle differentiation, Pax3/7 (E-10: sc-365613), MyoD (M-318: sc-760), myogenin (F5D: sc-12732), and myosin heavy chain (H-300: sc-20641) (Santa Cruz Biotechnology, Dallas, TX) were used. To detect IGFBP-6, antibody H-70: sc-13094 (Santa Cruz Biotechnology, Dallas, TX) was used. To detect IGF-2, antibody H-103: sc-5622 (Santa Cruz Biotechnology, Dallas, TX) was used. For loading control, pan-Actin Ab-5 (Thermo Fisher Scientific, Fremont, CA) was used. The secondary antibodies used for immunoblotting were HRP-conjugated goat anti-rabbit (#170-6515) or anti-mouse (#170-6516) (Bio-Rad, Hercules, CA) or donkey anti-goat antibody (Santa Cruz Biotechnology, Dallas, TX). The secondary antibodies used for immunocytochemistry were green-Alexa 488 or red-Alexa 568 (Invitrogen, Mississauga, ON).

### 2.8. Quantification of IGFBP-6 and IGF-2 by Enzyme-Linked Immunosorbent Assay (ELISA)

Human IGFBP-6 (RayBiotech®, Burlington, ON) and IGF-2 (ALPCO, Salem, NH) ELISA kits were used to measure the amount of IGFBP-6 and IGF-2 secreted into PMSC conditioned media. Standards and samples were loaded into the wells and the immobilized antibody bound IGFBP-6 or IGF-2 present in the sample. The wells were washed and biotinylated anti-human antibody was added. After washing, HRP-conjugated streptavidin was added; then, a TMB substrate solution was used to develop a blue color in proportion to the amount of IGFBP-6 or IGF-2 bound. The Stop Solution changes color from blue to yellow, and the intensity was measured at 450 nm using Multiskan Ascent plate reader and analysis software [26].

### 2.9. Aldehyde Dehydrogenase (ALDH) Activity

ALDH activity, a conserved progenitor cell function, was assessed by flow cytometry. Using the Aldefluor™ assay (Stem Cell Technologies, Vancouver, BC), as per the manufacturer's instructions. Briefly, 5 *μ*L of activated Aldefluor reagent was added to 1 mL of cell suspension and incubated for 45 minutes at 37°C. Cells were washed and resuspended in 500 *μ*L of ice-cold Aldefluor assay buffer and ALDH activity was measured using flow cytometry. As a negative control, Aldefluor DEAB reagent was used [26]. Samples were run in triplicate.

### 2.10. Statistical Analysis

All experiments were performed in triplicate using the cells from 3 PMSC samples (*N* = 3). GraphPad Prism Software 5.0 was used to generate all graphs and analyses. A two-way ANOVA followed by a Bonferroni's multiple comparison test or a one-way ANOVA followed by a Student's *t*-test was used to calculate significant differences when *P* < 0.05. Graphic representation values are presented as mean ± SEM (shown as variance bars).

## 3. Results

### 3.1. IGF-1 Affects PMSC Multipotency and Differentiation into Skeletal Muscle

**Figure 1:**
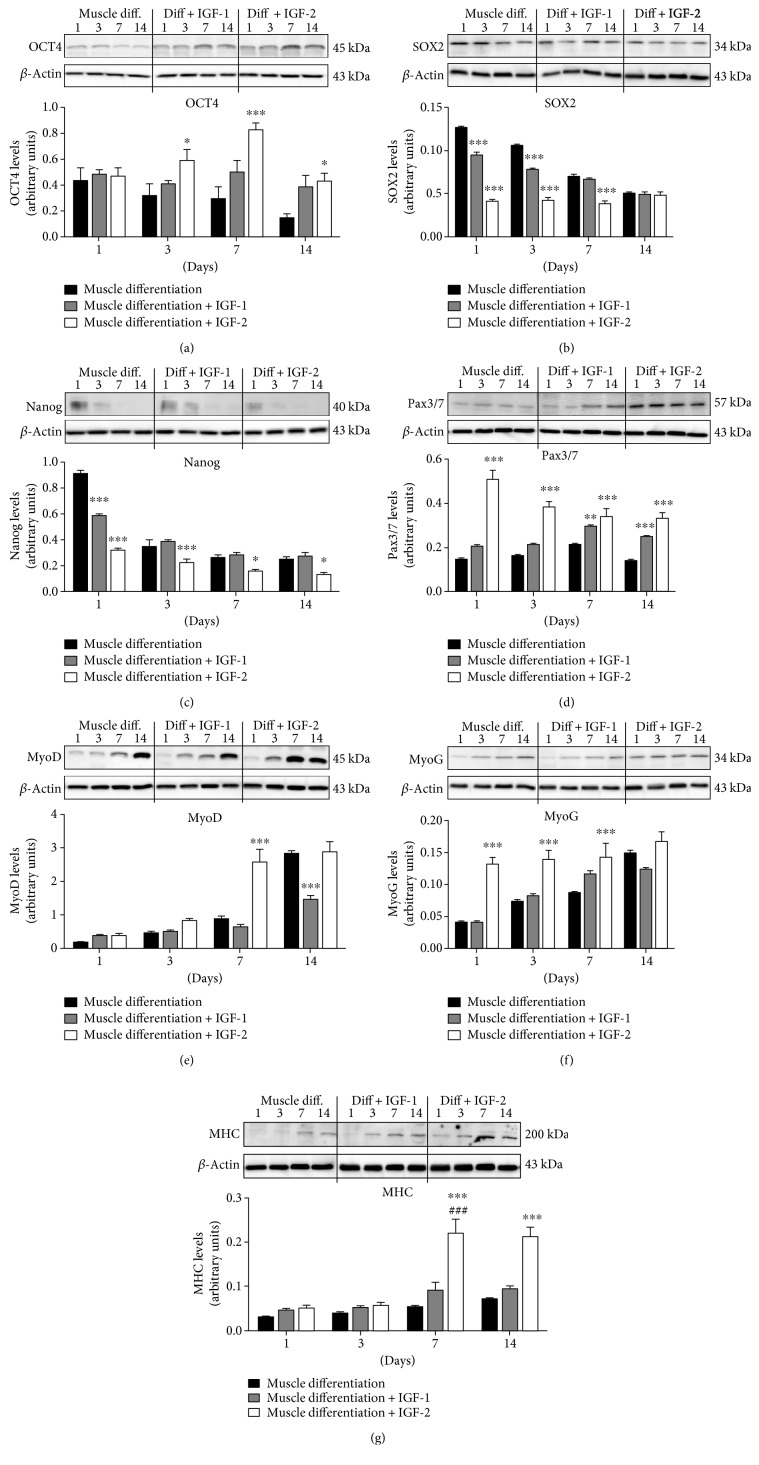
IGF-1 and IGF-2 supplementation differently affected levels of pluripotency-associated and muscle differentiation markers in PMSC. (a) IGF-1 treatment had no effect on OCT4 levels, while IGF-2 treatment increased OCT4 levels at 3, 7, and 14 days compared to muscle differentiation alone. (b) SOX2 and (c) Nanog levels were decreased after IGF-1 treatment at early time points only, whereas IGF-2 treatment decreased SOX2 levels until day 7 and at all time points for Nanog. (d) Pax3/7 levels were increased at 7 and 14 days and did not decrease after IGF-1 treatment, while IGF-2 treatment increased Pax3/7 levels at all time points but decreasing with time. (e) MyoD, (f) MyoG, and (g) MHC protein levels did not change with IGF-1 supplementation, except for a decrease in MyoD levels at day 14. In contrast, IGF-2 treatment increased muscle differentiation marker levels at all time points. Protein levels were quantified by densitometry and normalized to *β*-actin. Data is presented as the mean ± SEM of 3 independent experiments. Two-way ANOVA with Bonferroni's multiple comparison test was performed to determine ^∗^*P* < 0.05, ^∗∗^*P* < 0.01, and ^∗∗∗^*P* < 0.001 comparing control to muscle differentiation conditions, or ^###^*P* < 0.001 comparing the same treatment over time.

To test the effects of extracellular IGF-1 on PMSC multipotency and differentiation into muscle cells, IGF-1 was supplemented to muscle differentiation media (100 ng/mL) every third day for up to 14 days. OCT4 levels were not changed by IGF-1 supplementation (Figure 1(a)), while both SOX2 (Figure 1(b)) and Nanog (Figure 1(c)) levels were reduced at early time points compared to unsupplemented muscle differentiation conditions. In contrast, IGF-1 supplementation increased Pax3/7 levels at 7 and 14 days suggesting PMSC commitment to the muscle lineage (Figure 1(d)). Finally, IGF-1 treatment decreased the levels of muscle-specific differentiation marker, MyoD, at day 14 (Figure 1(e)), but did not change MyoG and MHC levels (Figures 1(f) and 1(g)), compared to PMSCs cultured under unsupplemented muscle differentiation conditions.

**Figure 2:**
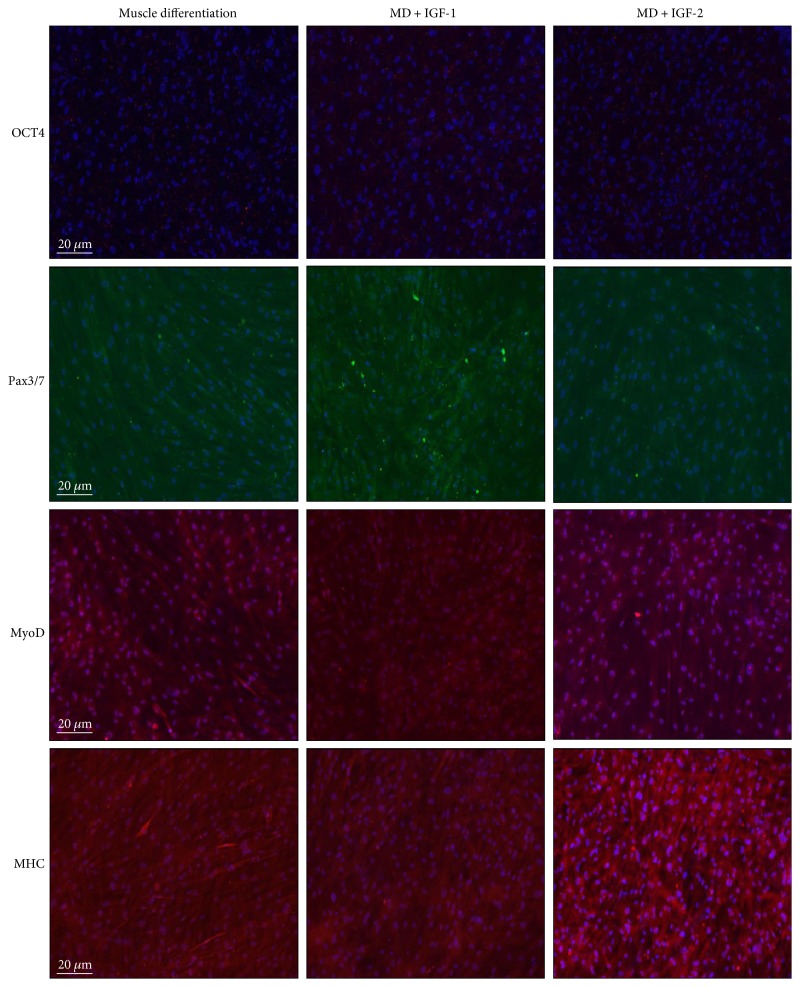
PMSCs treated with IGF-2 showed qualitatively increased immunofluorescence for MHC at 14 days. Compared to PMSCs cultured under muscle differentiation alone, PMSCs treated with IGF-1 or IGF-2 showed similar OCT4 immunoreactivity (red-Alexa, *λ* = 568 nm). However, IGF-1 supplementation increased Pax3/7 IR (green-Alexa, *λ* = 488 nm), decreased MyoD IR, with no change in MHC IR (red-Alexa, *λ* = 568 nm). In contrast, IGF-2 treatment did not alter Pax3/7 IR (green-Alexa, *λ* = 488 nm) or MyoD IR, but increased MHC IR (red-Alexa, *λ* = 568 nm). Nuclei, stained with Hoechst dye (blue, *λ* = 340 nm). Immunocytochemistry was performed in triplicate with each antibody.

Immunocytochemistry analyses at day 14 revealed that PMSCs treated with IGF-1 showed qualitatively increased Pax3/7, decreased MyoD, and no change in OCT4 and MHC immunoreactivity (Figure 2), which was in agreement with the analyses by Western blots. Moreover, IGF-1 treatment significantly increased the total number of cells compared to unsupplemented controls (Supplementary Figure 1).

**Figure 3:**
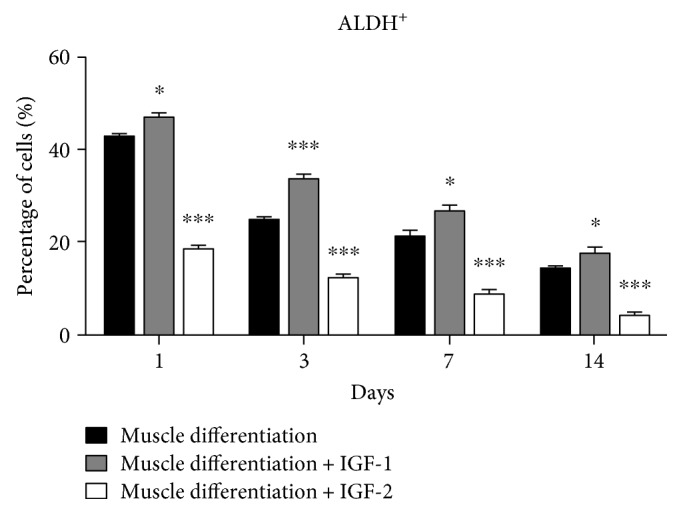
PMSCs cultured under skeletal muscle differentiation conditions treated with IGF-1 showed an increased frequency of cells with high ALDH activity, while cells treated with IGF-2 showed a decreased frequency. Compared to PMSCs under muscle differentiation conditions, cells treated with IGF-1 showed increased frequency of cells with high ALDH activity, while cells treated with IGF-2 showed decreased frequency of cells with high ALDH activity. Data is presented as the mean ± SEM of 3 independent experiments. Two-way ANOVA with Bonferroni's multiple comparison test was performed to determine ^∗^*P* < 0.05 and ^∗∗∗^*P* < 0.001.

We also tested PMSCs for ALDH activity to determine the frequency of cells that maintained high ALDH activity, a conserved phenotype of early progenitor cells in multiple lineages [27–29]. Under muscle differentiation conditions, IGF-1 treatment increased the frequency of cells with high ALDH activity at all time points compared to PMSCs under muscle differentiation alone (Figure 3 and Supplementary Figures 2A to 2D) suggesting that extracellular IGF-1 treatment prolonged progenitor cell phenotype in PMSCs cultured under muscle differentiation conditions.

### 3.2. IGF-2 Affects PMSC Multipotency and Differentiation into Skeletal Muscle

The impact of IGF-2 in PMSC multipotency and differentiation into skeletal muscle was investigated by adding IGF-2 (100 ng/mL) to the muscle differentiation media. Using Western blots, we demonstrated that IGF-2 supplementation increased the protein levels of pluripotency-associated marker OCT4 at days 3, 7, and 14 compared to PMSCs cultured in muscle differentiation media alone (Figure 1(a)), whereas other pluripotency-associated markers SOX2 (Figure 1(b)) and Nanog (Figure 1(c)) were decreased. In contrast, IGF-2 supplementation increased the protein levels of the muscle lineage commitment marker Pax3/7 and the levels of muscle differentiation markers MyoD, MyoG, and MHC compared to untreated controls (Figures 1(d)to 1(g)).

Using immunocytochemistry, PMSCs cultured under muscle differentiation conditions supplemented with IGF-2 showed, qualitatively, no change in OCT4, Pax3/7, and MyoD immunoreactivity (Figure 2). In contrast, expression of muscle differentiation marker MHC was increased (Figure 2) at 14 days after IGF-2 treatment compared to untreated controls, with fewer cells per field (Supplementary Figure 1) suggesting that IGF-2 enhanced the terminal muscle differentiation process.

Moreover, IGF-2 supplementation of PMSCs under muscle differentiation conditions decreased the frequency of cells with high ALDH activity compared to PMSCs under untreated muscle differentiation condition at all time points (Figure 3 and Supplementary Figures 2A to 2D), suggesting that IGF-2 promoted the differentiation of PMSCs into skeletal muscle.

### 3.3. Extracellular IGFs Altered IGFBP-6 Levels

**Figure 4:**
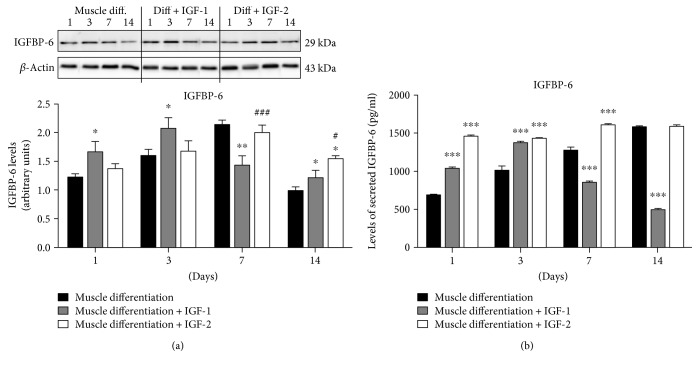
IGFBP-6 expression is altered by IGF-1 and IGF-2 treatment in PMSCs cultured under skeletal muscle differentiation conditions. (a) IGFBP-6 levels were increased by IGF-1 treatment at days 1 and 3 compared to PMSCs under muscle differentiation alone; however, they were reduced at days 7 and 14. IGF-2 treatment did not change IGFBP-6 levels until day 14 when they were increased compared to controls. IGFBP-6 levels were quantified by densitometry and normalized to *β*-actin. (b) IGFBP-6 secretion into conditioned media was increased with both IGF-1 (days 1 and 3) and IGF-2 (days 1 to 7) compared to controls. However, secreted IGFBP-6 levels were significantly decreased at 7 and 14 days with IGF-1 treatment, and remained increased with IGF-2 treatment. Data is presented as the mean ± SEM of 3 independent experiments. Two-way ANOVA with Bonferroni's multiple comparison test was performed to determine ^∗^*P* < 0.05, ^∗∗^*P* < 0.01, and ^∗∗∗^*P* < 0.001 compared to control (PMSCs under muscle differentiation), or ^#^*P* < 0.05 and ^###^*P* < 0.001 comparing between IGF-1 and IGF-2 treatments.

Using Western blots, we showed increased cellular IGFBP-6 levels with IGF-1 treatment at early time points, which coincided with delayed muscle commitment as indicated by higher levels of Pax3/7 levels (Figure 1(d)). In contrast, IGF-2 did not increase IGFBP-6 levels until day 14 compared to PMSCs under muscle differentiation only (Figure 4(a)). When compared to PMSCs treated with IGF-1, IGF-2 treatment increased IGFBP-6 levels at 7 and 14 days (Figure 4(a)) suggesting that IGF-2 treatment stimulated IGFBP-6 synthesis after PMSC commitment to the muscle lineage, whereas IGF-1 effect on IGFBP-6 synthesis occurred before PMSC commitment to muscle.

To investigate the effects of IGF-1 and IGF-2 on IGFBP-6 secretion into conditioned media under muscle differentiation conditions, IGFBP-6 in the PMSC media was measured using ELISA. With IGF-1 treatment, IGFBP-6 concentration was increased at early time points (days 1 and 3) and decreased at later time points (days 7 and 14) compared to control PMSCs under muscle differentiation conditions (Figure 4(b)). In contrast, IGF-2 treatment increased IGFBP-6 secretion throughout differentiation until day 14 (Figure 4(b)). Therefore, both IGFs increased IGFBP-6 synthesis by PMSCs under muscle differentiation conditions with the effect of IGF-1 short lived and IGF-2 for a long duration.

### 3.4. Extracellular IGFBP-6 Maintains Muscle Differentiation of PMSCs in the Absence of IGF-2

**Figure 5:**
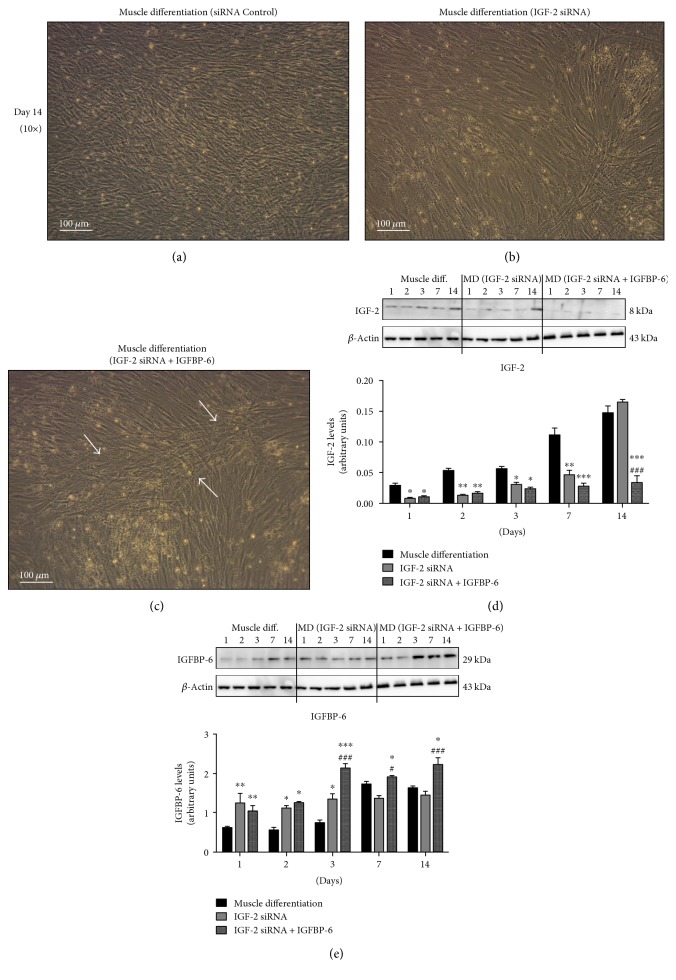
IGF-2 knockdown with siRNA inhibited PMSC differentiation into skeletal muscle and adding extracellular IGFBP-6 with the treatment helped rescue muscle compaction. (a) Compared to PMSCs under muscle lineage differentiation conditions with siRNA scrambled control, (b) PMSCs under muscle differentiation conditions treated with IGF-2 siRNA showed less skeletal muscle compaction at 14 days (10x). (c) Extracellular IGFBP-6 supplementation with IGF-2 knockdown enhanced the PMSC muscle compaction at 14 days compared to IGF-2 siRNA. The white arrows indicate muscle compaction. (d) IGF-2 levels by PMSCs treated with IGF-2 siRNA under differentiation conditions were reduced and returned to control levels by day 14, while adding IGFBP-6 with IGF-2 knockdown maintained lower IGF-2 levels. (e) IGFBP-6 protein levels increased until day 3 with IGF-2 knockdown compared to siRNA scrambled control. IGFBP-6 addition with IGF-2 knockdown increased IGFBP-6 protein levels at each time point. Protein levels were quantified by densitometry and normalized to *β*-actin. Data is presented as the mean ± SEM of 3 independent experiments. Two-way ANOVA with Bonferroni's multiple comparison test was performed to determine ^∗^*P* < 0.05, ^∗∗^*P* < 0.01, and ^∗∗∗^*P* < 0.001 compared to scrambled siRNA control, or ^#^*P* < 0.05 and ^###^*P* < 0.001 compared to IGF-2 siRNA.

Previous studies in the Han Laboratory showed that during PMSC differentiation into skeletal muscle, IGF-2 secretion was significantly increased as compared to control (10% FBS), confirming that developing muscle cells express IGF-2 which is actively secreted [26]. To evaluate the role of endogenous IGF-2 on PMSC differentiation into skeletal muscle, IGF-2 mRNA was silenced using siRNA treatment every 3 days for up to 14 days. PMSCs with IGF-2 knockdown showed less compact muscle morphology compared to control (scrambled siRNA) at day 14 (Figures 5(a) and 5(b)). The addition of IGFBP-6 together with IGF-2 siRNA permitted PMSCs compact muscle morphology at 14 days (Figures 5(b) and 5(c)). As expected, IGF-2 levels were decreased by IGF-2 knockdown compared to scrambled siRNA control. However, IGF-2 levels were equivalent to control levels at day 14 although IGF-2 siRNA was administered every 3 days (Figure 5(d)) indicating that siRNA-treatment was transient. Furthermore, IGF-2 levels remained significantly low at all of the time points by the addition of IGFBP-6 with IGF-2 knockdown and did not return to control levels (Figure 5(d)), suggesting an interaction between IGF-2 and IGFBP-6.

After IGF-2 knockdown, IGFBP-6 levels were increased until day 3 compared to scrambled siRNA control (Figure 5(e)), suggesting greater availability of IGFBP-6 demonstrated by an increase in secreted IGFBP-6 (Supplementary Figure 3). As expected, IGFBP-6 protein levels were significantly increased at each time point during IGF-2 siRNA treatment alongside extracellular IGFBP-6 supplementation compared to controls (Figure 5(e)).

**Figure 6:**
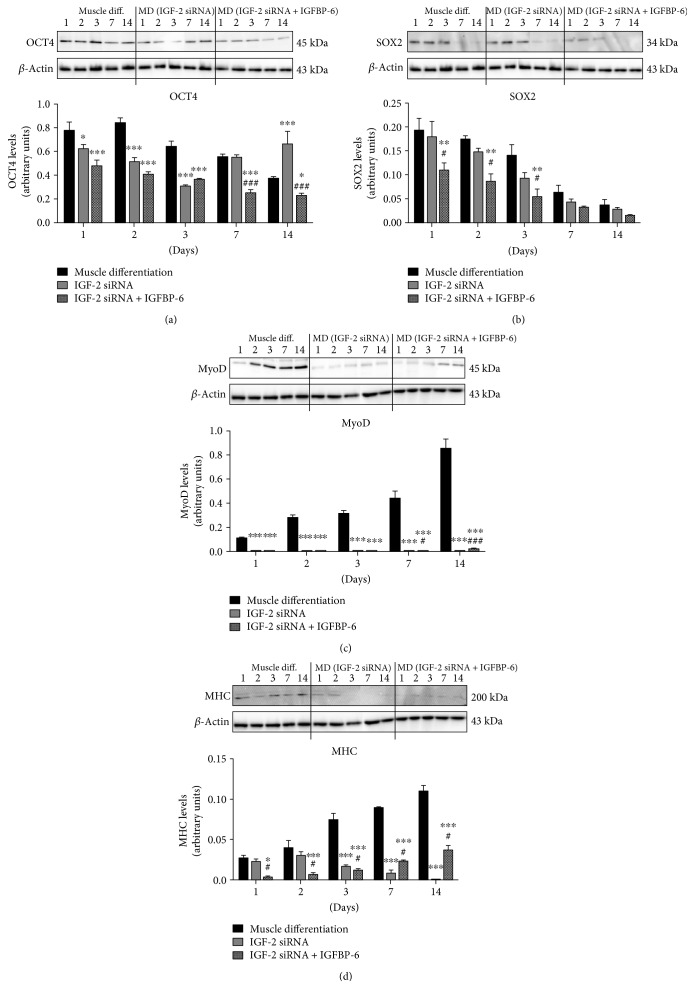
IGF-2 knockdown decreased muscle differentiation markers, while adding IGFBP-6 partially increased the markers. (a) OCT4 levels were reduced with IGF-2 knockdown until day 3 and increased at day 14. Adding IGFBP-6 alongside IGF-2 siRNA reduced OCT4 protein levels at all-time points compared to siRNA scrambled control. In contrast, (b) SOX2 levels did not change with IGF-2 knockdown. But when IGFBP-6 was added with IGF-2 silencing, the levels were reduced from 1 to 3 days compared to control and IGF-2 knockdown. Protein levels of muscle differentiation markers: (c) MyoD and (d) MHC decreased with IGF-2 siRNA and IGFBP-6 supplementation increased the levels. Protein levels were quantified by densitometry and normalized to *β*-actin. Data is presented as the mean ± SEM of 3 independent experiments. Two-way ANOVA with Bonferroni's multiple comparison test was performed to determine ^∗^*P* < 0.05, ^∗∗^*P* < 0.01, and ^∗∗∗^*P* < 0.001 compared to scrambled siRNA control, or ^#^*P* < 0.05 and ^###^*P* < 0.001 compared to IGF-2 siRNA.

Concurrent with IGF-2 knockdown, we observed a decrease in pluripotency-associated marker OCT4 levels until day 3 with an increase at day 14 compared to scrambled siRNA control (Figure 6(a)). In contrast, SOX2 levels did not change (Figure 6(b)). Furthermore, the addition of IGFBP-6 with IGF-2 knockdown reduced both OCT4 (Figure 6(a)) and SOX2 (Figure 6(b)) levels. The protein levels of muscle lineage differentiation markers MyoD (Figure 6(c)) and MHC (Figure 6(d)) were decreased significantly after IGF-2 knockdown indicating a critical role for IGF-2 in PMSC differentiation into skeletal muscle. In contrast, IGFBP-6 supplementation alongside IGF-2 knockdown significantly increased MyoD and MHC levels at 7 and 14 days compared to siRNA scrambled control or IGF-2 knockdown alone (Figures 6(c) and 6(d)).

**Figure 7:**
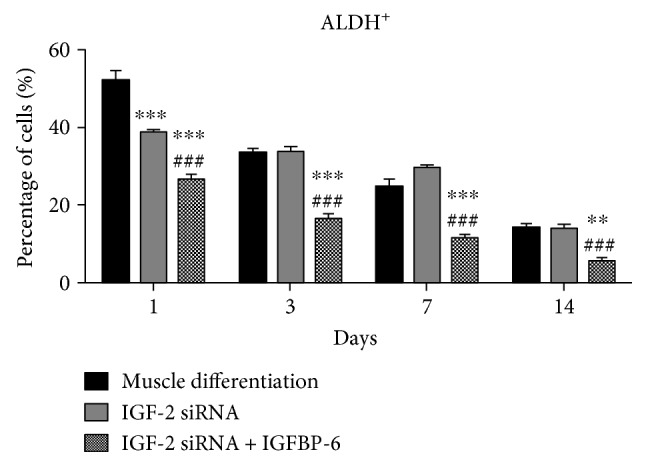
IGF-2 knockdown and extracellular IGFBP-6 addition in PMSCs under muscle differentiation conditions decreased the frequency of cells with high ALDH activity. Compared to PMSCs under muscle differentiation conditions (siRNA scrambled control), cells treated with IGF-2 siRNA showed decreased frequency of cells with high ALDH activity at day 1, while adding extracellular IGFP-6 with IGF-2 siRNA showed decreased frequency of cells with high ALDH activity at each time point. Data is presented as the mean ± SEM of 3 independent experiments. Two-way ANOVA with Bonferroni's multiple comparison test was performed to determine ^∗∗^*P* < 0.01, and ^∗∗∗^*P* < 0.001 compared to scrambled siRNA control, or ^###^*P* < 0.001 compared to IGF-2 siRNA.

Knockdown of IGF-2 expression in PMSCs significantly decreased the abundance of cells with high ALDH activity compared to control (scrambled siRNA) at day 1 (Figure 7 and Supplementary Figure 4). In contrast, the addition of IGFBP-6 together with IGF-2 knockdown further decreased the abundance of cells with high ALDH activity at all time points compared to siRNA scrambled control or IGF-2 siRNA treatment (Figure 7 and Supplementary Figure 4). Collectively, these data suggest that IGFBP-6 reduced progenitor cell phenotype under muscle differentiation conditions and maintained the differentiation of PMSCs towards skeletal muscle in the absence of IGF-2.

## 4. Discussion

It is believed that if stem cells are to be used successfully in cell-based therapies for specific diseases, they must be initiated towards a progenitor cell of a desired lineage (e.g., skeletal muscle for therapy of muscular dystrophy) [30]. In addition, adequate cell numbers will be needed for effective therapy. Human placenta, which is usually discarded following birth, is a potential source of adult mesenchymal stem cells with functional capacity similar to bone marrow [31–34]. PMSCs also demonstrate low tumorigenicity with higher immunotolerance after transplantation, making them an ideal cell type for tissue regeneration therapies [35, 36].

The IGF system is important for muscle development, growth, regeneration, and differentiation [10, 11, 13, 37, 38]. IGF-2 plays an important role during C2C12 differentiation and is considered the main myogenic factor in myoblast cells [39]. In C3H 10T1/2 fibroblasts converted to myoblasts by transfection of MyoD transgene, there was an increase in the mRNA expression and protein levels of IGF-2 during the differentiation stage [16]. In this study, we showed that IGF-2 is synthesized and secreted into the extracellular space by PMSCs during muscle differentiation and the highest levels are expressed by fully differentiated muscle cells. Taken together, these findings show the important role of the IGFs, particularly IGF-2, in muscle differentiation as autocrine/paracrine factors.

IGF-binding proteins act as carriers for IGF-1 and IGF-2 in the circulation, facilitating ligand delivery to specific tissues and controlling access to the IGF receptors [2, 3, 40]. Also, IGFBPs are expressed by many cell types, including skeletal muscle, and have been demonstrated to have functions that are dependent or independent of IGF binding [4]. IGFBPs are expressed by developing muscle cells and are important in myogenesis [40, 41]. In osteoblasts, IGFBP-6 has been shown to modulate cell growth by reducing the bioavailability of IGF-2 in the bone microenvironment [42]. When L6E9 cells (a myoblast cell line used to study late myogenesis) are stimulated with IGF-1, these cells initiate a proliferative response. During this time of rapid cell division, the myogenic regulatory factors are inhibited. Approximately 30 hours later, there is a stimulation of myogenin expression [43]. In mouse C2C12 cells and C2 satellite cell line, there are greater levels of IGF-2 mRNA than IGF-1, 2000-fold to 20-fold, respectively [12, 19, 44]. Our study is one of the first to show the effects of IGF-1 and IGF-2, in combination with IGFBP-6, on human PMSC differentiation into skeletal muscle *in vitro*.

The aim of this study was to characterize the effects of IGFs on the differentiation of PMSCs into skeletal muscle and to delineate their interactions with IGFBP-6 in the differentiation process. We showed previously that IGF-2 secretion into the condition media was increased during and at the completion of muscle differentiation indicating that the synthesis of IGF-2 increased as the cells became more differentiated [26]. Moreover, increased IGFBP-6 in the PMSC microenvironment is expected to reduce the bioavailability of IGF-2 due to its high affinity for the peptide, confirmed by IGF-2 ELISA [26].

**Figure 8:**
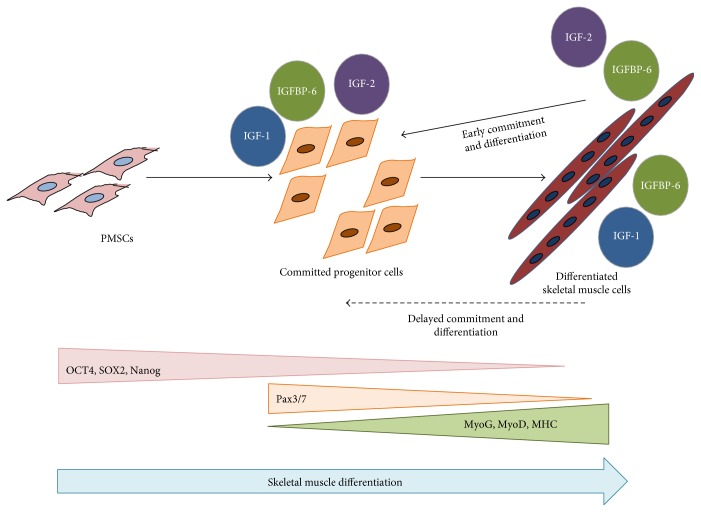
Model of IGFs and IGFBP-6 functions during PMSCs myogenesis. During myogenesis, PMSCs lose pluripotency-associated markers (OCT4, SOX2, and Nanog) and gain muscle commitment marker (Pax3/7) that decreased as muscle differentiation markers increase (MyoG, MyoD, and MHC). Committed and differentiated muscle cells expressed IGF-1, IGF-2, and IGFBP-6. Extracellular IGF-1 increased IGFBP-6 protein levels before PMSC muscle commitment, resulting in a delayed PMSC muscle commitment and differentiation. However, IGF-2 extracellular increase resulted in an increase in IGFBP-6 after commitment to the muscle lineage, resulting in full muscle lineage differentiation. Increased IGF-2 and IGFBP-6 levels also had a positive effect on OCT4 levels, but SOX2 and Nanog levels were decreased.

In this study, we showed that IGF-1 can promote an early increase in IGFBP-6 expression before PMSCs commit to the muscle lineage, a requirement that delayed muscle lineage commitment. This was confirmed by the increased levels of muscle commitment marker (Pax3/7) and decreased muscle differentiation marker (MyoD) after IGF-1 treatment. On the other hand, IGF-2 treatment increased both IGBP-6 and OCT4 levels. These results are in agreement with our previous studies [26], showing IGFBP-6 positive effects on OCT4 levels. Also, the muscle differentiation markers (MyoD, MyoG, and MHC) were increased at later time points with IGF-2 treatment, confirming that IGF-2 enhanced PMSC muscle differentiation, unlike IGF-1 (Figure 8). Increased OCT4 levels occurred alongside a decrease in SOX2 and Nanog levels which is expected in a mesodermal differentiation [45]; OCT4 is needed for differentiation as it supports downregulating pluripotency, and when deficient, cells are not able to differentiate [46].

IGF-1 and IGF-2 stimulate both proliferation and terminal differentiation of many tissues in developing embryos and adults. IGFBP-6 has a significantly higher affinity (~70–100-fold) for IGF-2 than IGF-1 [19–22]. Previous studies in C2C12 cells show that as muscle differentiation progressed, IGF-2 stimulated its own expression and inhibited IGF-1 expression in a time- and dose-dependent manner [12]. In our study, there was a decrease in the amount of secreted IGF-2 to the media after IGF-1 treatment in PMSCs under muscle differentiation conditions, indicating that an intricate balance between IGF-1 and IGF-2 expression exists in the niche during myogenesis from PMSCs (Supplementary Figure 5). Further investigation will be conducted to delineate these effects.

Studies in various cell lines have shown mostly inhibitory action of IGFBP-6 mainly via IGF-2-dependent mechanism. In L6A1 myoblasts, recombinant human IGFBP-6 inhibited muscle differentiation stimulated by IGF-2 in a dose-dependent manner but had no effect on IGF-1 induced differentiation [47]. Moreover, IGFBP-6 expression has been previously associated with nonproliferative states and inhibition of IGF-2 dependent tumor cell growth in rhabdomyosarcoma, neuroblastoma, and colon cancer [48]. More specifically, neuroblastoma cells undergo a decrease in both cell proliferation and tumorigenic potency as a result of exogenous IGFBP-6 expression as IGFBP-6 sequesters IGF-2 preventing a mitogenic response in tumor cells [49, 50]. Previous reports on the effects of IGFs on muscle differentiation used mouse cell lines [8, 12, 19, 39, 41, 44], thus our study is one of the first to show the effects of IGF-dependent functions of IGFBP-6 on human MSC differentiation into skeletal muscle *in vitro*.

In this study, we focused on the role of IGFBP-6 and IGF-2 in PMSCs under muscle differentiation conditions. We showed that IGFBP-6 was required for PMSC differentiation into skeletal muscle and modulated both multipotency and muscle markers levels, as well as IGF-2 secretion. When IGF-2 was knocked down using siRNA, myogenesis was inhibited, and adding IGFBP-6 helped recover the muscle differentiation process, supported by muscle morphology and differentiation markers. Previous studies [48–50] are in agreement with our findings, as IGF-2 levels are significantly reduced with the increase in extracellular IGFBP-6.

Placenta development is dependent on the IGF system, including IGF-1 and IGF-2 [51]. The importance of IGFs in the human placenta is well defined in mediating growth and differentiation of the different cells of the chorionic villi [5, 52]. In the human placenta, IGF-2 mRNA is expressed in the villous mesenchymal core, where PMSCs reside [51]. IGF-2 also plays a role in the placenta at the early gestation, whereas IGF-1 is associated in later gestation [53]. Results from this study showed that IGF-1 and IGF-2 had different effects on PMSC differentiation into skeletal muscle in a time-dependent manner. The role of IGF-1 and IGF-2 in PMSC differentiation into skeletal muscle is not clearly defined and this study is the first to show the different effects between IGF-1 and IGF-2.

**Figure 9:**
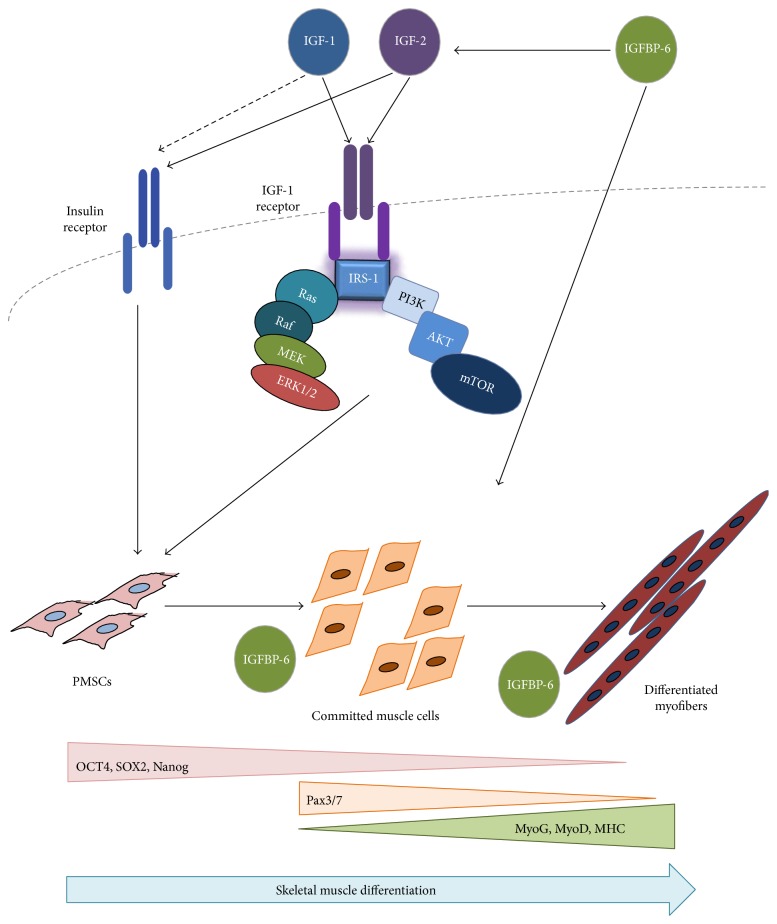
PMSC differentiation into skeletal muscle using the insulin-like growth factor system. PMSCs isolated from the chorionic villus of preterm human placenta expressed high levels of pluripotency-associated markers under normal growth conditions (10% FBS). As these cells differentiated into skeletal muscle, the levels of these markers decreased, and the cells committed to the muscle lineage, indicated by Pax3/7 expression. Once committed to differentiation, PMSCs subsequently decreased Pax3/7 expression and increased muscle differentiation markers (MyoG, MyoD, and MHC) as myoblasts aligned and fuse to form multinucleated myofibers. IGF-1 and IGF-2 binds to the IGF-1R and activates its intrinsic tyrosine kinase activity resulting in signaling that accelerated muscle differentiation via downstream signals including PI3K-AKT-mTOR and the RAF-MEK-ERK (MAPK) pathway. Due to IGFBP-6 intracellular and extracellular locations, IGFBP-6 demonstrated both IGF-dependent and IGF-independent effects on PMSC muscle differentiation. Extracellular IGFBP-6 binds IGFs and enhances the muscle differentiation process through the IGF-1R, while intracellular IGFBP-6 directly impacted PMSC muscle differentiation through an unknown mechanism.

In conclusion, PMSC differentiation into skeletal muscle is regulated by IGFs. IGF-1 delays PMSC commitment towards the muscle lineage while IGF-2 enhanced myogenesis. IGFBP-6 is also required in this differentiation process, and extracellular addition of IGFBP-6 alongside IGF-2 inhibition sufficiently rescued PMSC muscle differentiation. Since IGFBP-6 has both intracellular as well as extracellular effects, we showed that the effects on muscle differentiation are both IGF-dependent and IGF-independent (particularly IGF-2) (Figure 9). Further investigation of the balance between IGFs and IGFBP-6 in PMSC myogenesis and delineating the receptors and signaling mechanisms governing muscle lineage development and regeneration will improve the success of cellular therapies in muscular dystrophies.

## Supplementary Materials

Supplementary Materials Supplementary Figure 1: PMSCs under muscle differentiation conditions treated with IGF-1 showed higher cell count, while IGF-2-treated PMSCs were lower compared to PMSCs in muscle differentiation alone. Data is presented as the mean ± SEM of 15 different fields from 3 independent experiments. One-way ANOVA followed by Student's t-test was performed to determine ^∗∗∗^*P* < 0.001 comparing to muscle differentiation. Supplementary Figure 2: Representative flow cytometry dot plots from 3 independent experiments showing the frequency of PMSC with high ALDH activity when cultured under muscle differentiation conditions with or without either IGF-1 or IGF-2 at (A) day 1, (B) day 3, (C) day 7, and (D) day 14. DEAB-treated controls were used to establish the ALDH gate. Supplementary Figure 3: IGFBP-6 levels secreted to the media increased with IGF-2 knockdown compared to muscle differentiation using ELISA. Data is presented as the mean ± SEM of 3 independent experiments. Two-way ANOVA with Bonferroni's multiple comparison test; ^∗∗∗^*P* < 0.001. Supplementary Figure 4: Representative flow cytometry dot plots from 3 independent experiments showing the frequency of PMSCs with high ALDH activity when cultured under muscle differentiation conditions with or without either IGF-2 siRNA or IGF-2 siRNA and extracellular IGFBP-6 at (A) day 1, (B) day 3, (C) day 7, and (D) day 14. DEAB-treated controls were used to establish the ALDH gate. Supplementary Figure 5: PMSCs treated with IGF-1 showed decreased IGF-2 levels secreted into the media compared to muscle differentiation. Data is presented as the mean ± SEM of 3 independent experiments. Two-way ANOVA with Bonferroni's multiple comparison test; ^∗^*P* < 0.05 and ^∗∗∗^*P* < 0.001.
