# Supplementary material for: Different Effects of Insulin-Like Growth Factor-1 and Insulin-Like Growth Factor-2 on Myogenic Differentiation of Human Mesenchymal Stem Cells
Source: Stem Cells Int. 2017 Dec 14;2017:8286248. doi: 10.1155/2017/8286248 (PMC5745708; doi:10.1155/2017/8286248)

**SUPPLEMENTARY FIGURES**

**Supplementary Figure 1: PMSCs under muscle differentiation conditions treated with IGF-1showed higher cell count, while IGF-2 treated PMSCs were lower compared to PMSCs in muscle differentiation alone.** Data is presented as the mean ± SEM of 15 different fields from 3 independent experiments. One-way ANOVA followed by a Student’s t-test was performed to determine ***P<0.001 comparing to muscle differentiation.

**Supplementary Figure 2:** Representative flow cytometry dot plots from 3 independent experiments showing the frequency of PMSC with high ALDH-activity when cultured under muscle differentiation conditions with or without either IGF-1 or IGF-2 at **(A)** day 1, **(B)** day 3 , **(C)** day 7 , and **(D)** day 14. DEAB treated controls were used to establish the ALDH gate.

**Supplementary Figure 3: IGFBP-6 levels secreted to the media increased with IGF-2 knockdown compared to muscle differentiation using ELISA.** Data is presented as the mean ± SEM of 3 independent experiments. Two-way ANOVA with Bonferroni’s multiple comparison test, ***=P<0.001.

**Supplementary Figure 4:** Representative flow cytometry dot plots from 3 independent experiments showing the frequency of PMSCs with high ALDH-activity when cultured under muscle differentiation conditions with or without either IGF-2 siRNA or IGF-2 siRNA and extracellular IGFBP-6 at **(A)** day 1, **(B)** day 3 , **(C)** day 7 , and **(D)** day 14. DEAB treated controls were used to establish the ALDH gate

**Supplementary Figure 5:** **PMSCs treated with IGF-1 showed decreased IGF-2 levels secreted into the media compared to muscle differentiation.** Data is presented as the mean ± SEM of 3 independent experiments. Two-way ANOVA with Bonferroni’s multiple comparison test, *P<0.05, ***=P<0.001.

**Supplementary Figure 1.**

**
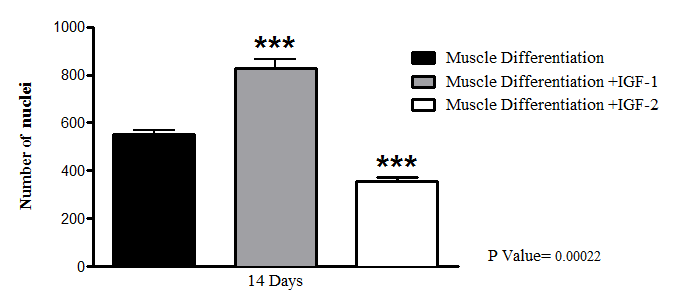
**

**Supplementary Figure 2.**

**
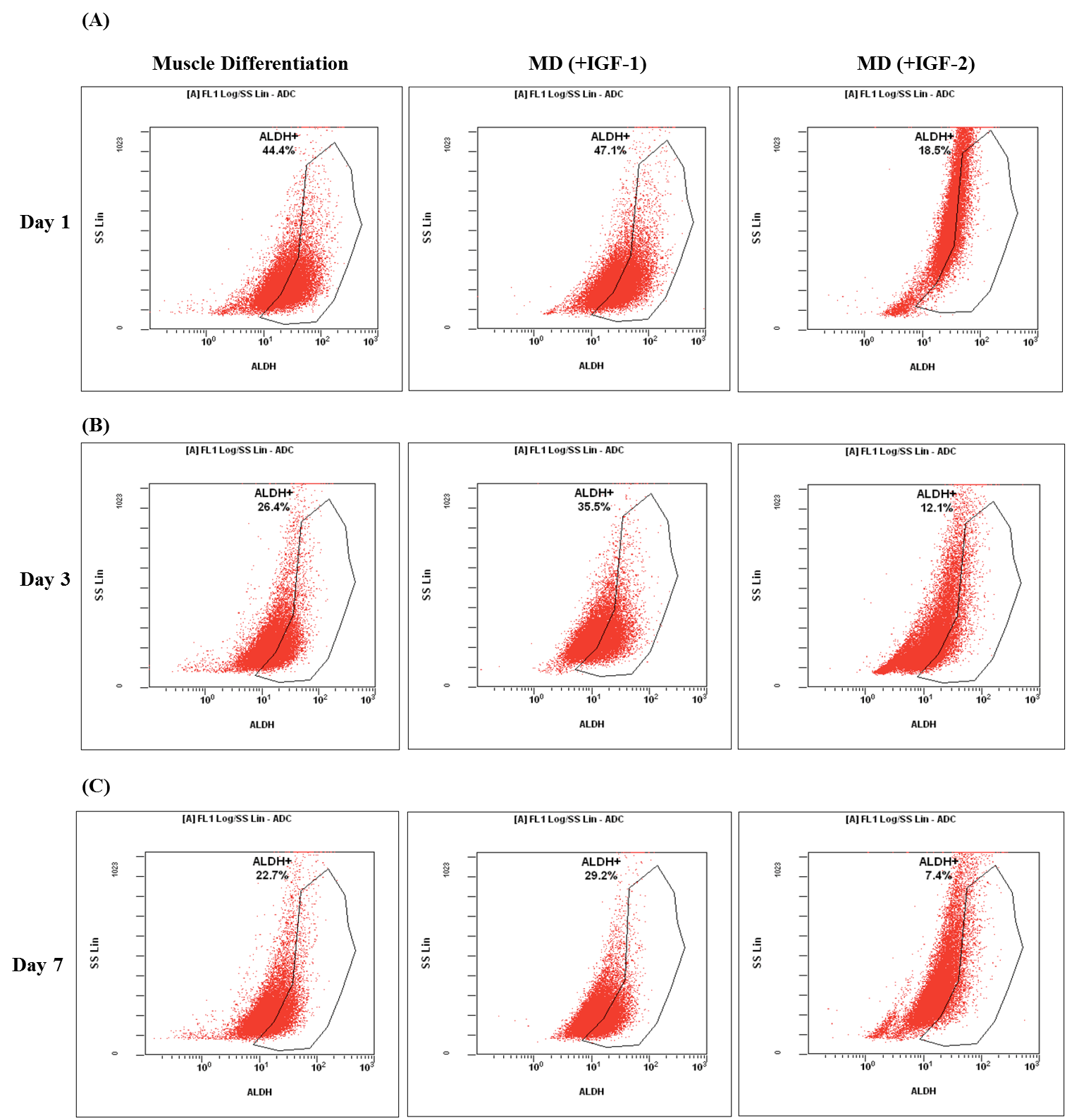
**

**
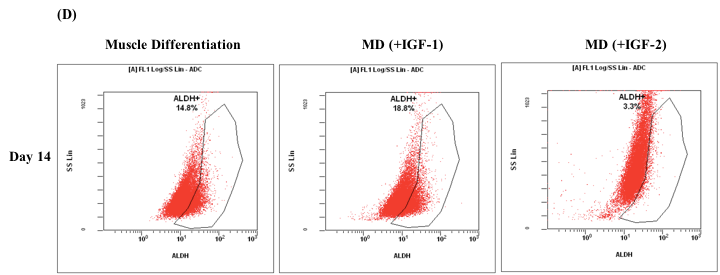
**

**Supplementary Figure 3.**


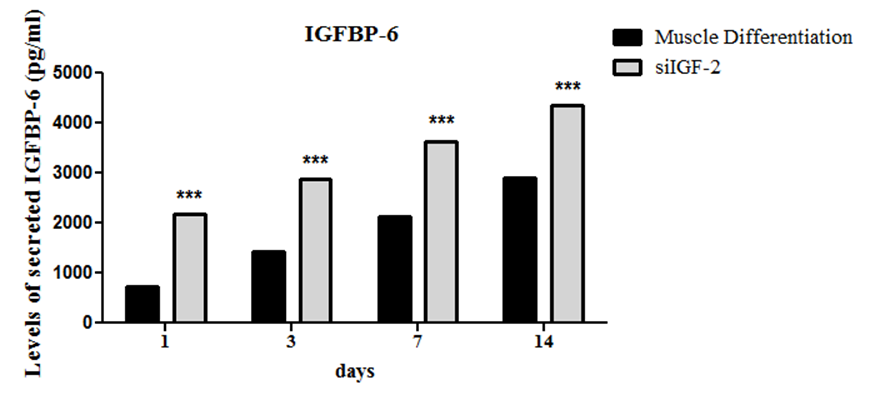


**Supplementary Figure 4.**

**
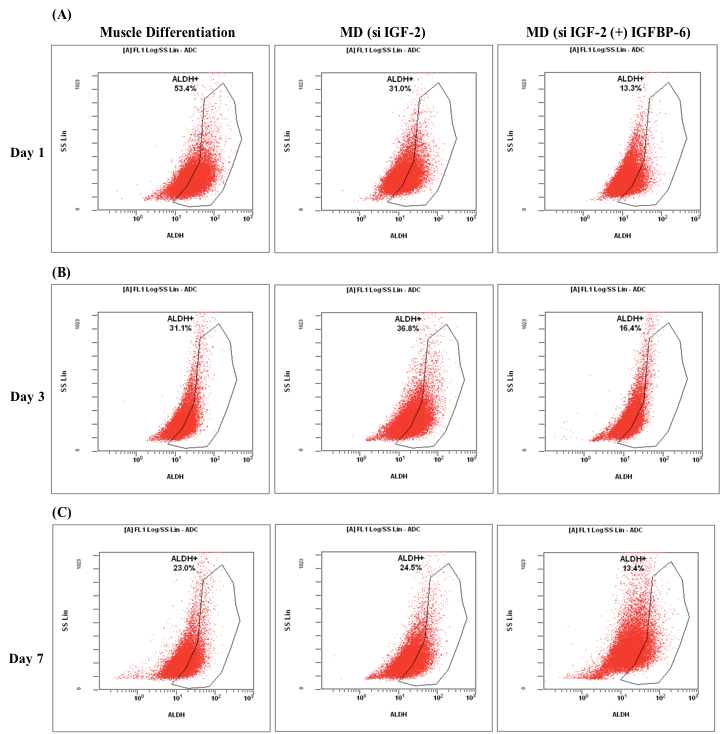
**

**
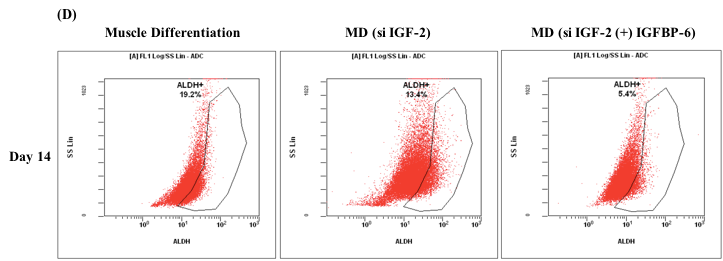
**

**Supplementary Figure 5.**


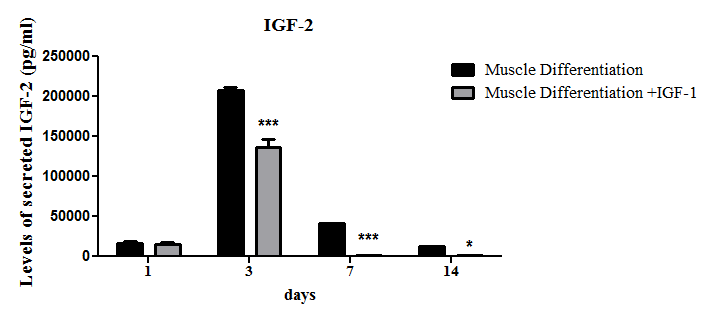

Supplement: Supplementary Materials — Supplementary Figure 1: PMSCs under muscle differentiation conditions treated with IGF-1 showed higher cell count, while IGF-2-treated PMSCs were lower compared to PMSCs in muscle differentiation alone. Data is presented as the mean ± SEM of 15 different fields from 3 independent experiments. One-way ANOVA followed by Student's t-test was performed to determine ∗∗∗P < 0.001 comparing to muscle differentiation. Supplementary Figure 2: Representative flow cytometry dot plots from 3 independent experiments showing the frequency of PMSC with high ALDH activity when cultured under muscle differentiation conditions with or without either IGF-1 or IGF-2 at (A) day 1, (B) day 3, (C) day 7, and (D) day 14. DEAB-treated controls were used to establish the ALDH gate. Supplementary Figure 3: IGFBP-6 levels secreted to the media increased with IGF-2 knockdown compared to muscle differentiation using ELISA. Data is presented as the mean ± SEM of 3 independent experiments. Two-way ANOVA with Bonferroni's multiple comparison test; ∗∗∗P < 0.001. Supplementary Figure 4: Representative flow cytometry dot plots from 3 independent experiments showing the frequency of PMSCs with high ALDH activity when cultured under muscle differentiation conditions with or without either IGF-2 siRNA or IGF-2 siRNA and extracellular IGFBP-6 at (A) day 1, (B) day 3, (C) day 7, and (D) day 14. DEAB-treated controls were used to establish the ALDH gate. Supplementary Figure 5: PMSCs treated with IGF-1 showed decreased IGF-2 levels secreted into the media compared to muscle differentiation. Data is presented as the mean ± SEM of 3 independent experiments. Two-way ANOVA with Bonferroni's multiple comparison test; ∗P < 0.05 and ∗∗∗P < 0.001. [file 8286248.f1.docx]
